# Multiple malignant primary tumors in dogs: an Italian registry-based epidemiological study

**DOI:** 10.3389/fvets.2026.1755025

**Published:** 2026-05-05

**Authors:** Maria Ines Crescio, Cristiano Cocumelli, Azzurra Carnio, Andrea Carvelli, Niccolò Fonti, Aitor Garcia-Vozmediano, Valentina Galietta, Claudia Eleni, Francesco Ingravalle, Elisabetta Manuali, Carmen Maresca, Francesca Millanta, Elisabetta Razzuoli, Paola Scaramozzino, Sara Simeoni, Marta Vascellari, Claudia Zanardello, Giuseppe Ru

**Affiliations:** 1 BeAR- Biostatistics and Risk Analysis-Istituto Zooprofilattico Sperimentale del Piemonte, Liguria e Valle d’Aosta, Torino, Italy; 2 Istituto Zooprofilattico Sperimentale del Lazio e Toscana, M. Aleandri, Roma, Italy; 3 Department of Comparative Biomedicine and Food Science, University of Padova, Legnaro, Italy; 4 Department of Veterinary Sciences, University of Pisa, Pisa, Italy; 5 Istituto Zooprofilattico Sperimentale dell’Umbria e delle Marche, Togo Rosati, Perugia, Italy; 6 CEROVEC-National Reference Center for Veterinary Oncology-Istituto Zooprofilattico Sperimentale del Piemonte, Liguria e Valle d’Aosta, Genova, Italy; 7 Istituto Zooprofilattico Sperimentale delle Venezie, Legnaro, Italy

**Keywords:** cancer, co-occurrence, dog, multiple tumors, risk

## Abstract

**Background:**

In human oncology, patients diagnosed with a primary malignant tumor are known to have an increased risk of developing subsequent malignancies. In veterinary medicine, however, registry-based studies investigating multiple malignant primary tumors (mMPTs) in dogs remain scarce. The increasing longevity of companion dogs, along with advances in diagnostics and therapies, underscores the importance of understanding the epidemiology and biological determinants of mMPTs.

**Objectives:**

This study aimed to assess the proportional occurrence and patterns of mMPTs in dogs diagnosed with malignant tumors, from four Regional Italian cancer registries integrated within the NILOV (Italian Network of Laboratories for Veterinary Oncology) between 2013 and 2024.

**Methods:**

A total of 16,618 canine cancer records were analyzed. Tumors were classified according to the Vet-ICD-O-Canine-1 system. Proportional morbidity ratios (PMRs) were estimated using multilevel mixed-effects Poisson regression models (random effects: subject and registry), stratified by sex, age, breed, and time, both including and excluding mammary gland tumors. Associations between tumor histotypes were evaluated using *χ*^2^ tests and network co-occurrence analysis (netCoin R package).

**Results:**

mMPTs were identified in 11.3% of oncologic dogs, with age and sex emerging as the associated factors. The risk increased progressively with age, peaking in dogs aged ≥12 years (PMR ≈ 7.3, 95% CI: 3.7–14.6). Females showed a modestly higher probability than males (PMR ≈ 1.3, 95% CI: 1.2–1.5). Breed had only a marginal effect, while the later registry period (2019–2024) showed a slight, likely artefactual decrease. Network analyses demonstrated that tumor co-occurrence is not random but organized in biologically meaningful clusters.

**Conclusion:**

This first large-scale, registry-based analysis of mMPTs in Italian dogs demonstrates that multiple malignancies are relatively common and influenced primarily by age and sex. The findings highlight the value of integrated cancer registries in veterinary oncology and support comparative studies aimed at uncovering shared mechanisms of oncogenesis across species.

## 1 Introduction

Multiple malignant primary tumors are defined as the occurrence, within the same subject, of distinct tumor histotypes, either at the same anatomical site or at different sites (1). In human oncology, it is well established that patients diagnosed with a primary malignancy face an increased risk of developing a second primary tumor (2). The incidence of second primary malignancies, in humans, has increased among cancer survivors due to improved diagnostics, treatments, and surveillance, with risk varying by cancer site and influenced by genetic, clinical, and environmental factors (3, 4). In humans in Italy, the mean standardized incidence ratio (SIR) for a second malignancy is 1.10 (95% CI: 1.09–1.10), with risk decreasing over time and influenced by age and sex being higher in younger patients and in females, who show an increased risk in 18 out of 30 cancer sites (5).

In veterinary medicine, despite a long-standing tradition of cancer registration in dogs and the establishment of several population-based registries worldwide, epidemiological studies specifically addressing multiple primary tumors in the canine population remain limited and outdated (6). Early investigations, such as that of Bender and coll. (7) using data from the Alameda and Contra Costa County tumor registry, reported a 1.8-fold increase in the risk of a second mammary gland malignancy and a 1.9-fold increase in the risk of a second skin tumor after a primary mammary tumor. Following malignant testicular tumors, they observed a 5.2-fold increased risk of malignant melanoma and a 3.7-fold increased risk of a second skin malignancy. More recently, Rebhun and coll. (8) described a prevalence of approximately 3% for multiple primary tumors (benign and malignant combined) in a case series of 53 dogs presented at the Colorado State University Veterinary Teaching Hospital, with no evidence of sex- or breed-related predisposition. Synchronous primary neoplasms have also been reported in case series based on diagnostic imaging, although these often lack consistent histological classification (9, 10).

The precedent for canine cancer surveillance in Italy was set with the establishment of the first population-based canine tumor registry in Genoa in 1985 (11). This was followed by the creation of numerous regional and provincial initiatives across the country, demonstrating a broad commitment to canine oncology data collection (12–24).

These registries are now integrated within the Italian Network of Laboratories for Veterinary Oncology (NILOV), a national network established in 2013 to centralize tumor diagnoses from multiple institutions and promote collaborative research. The NILOV database records detailed individual information, including breed, sex, neuter status, date of birth, date of diagnosis, geographic location, and anonymized owner identifiers. All the diagnosis were coded based on morphology and topography, using appropriately adapted ICD-O and ICD-X classification systems (25) standardized through consensus among participating pathologists (26). Since 2023, NILOV has adopted the vet-ICD-O-canine-1 classification system (27).

The increasing lifespan of companion dogs, together with advances in diagnostics and novel therapies, is expected to raise the number of animals living with a cancer diagnosis and to enhance the detection of multiple primary tumors (28, 29). Studying these cancers is therefore crucial: for practitioners, to guide patient follow-up with quantitative data stratified by sex, age, and tumor site and to design appropriate therapeutic protocols while avoiding the stimulation of oncogenesis in tumor histotypes that are biologically associated (30); for pet owners, to understand whether their dog’s risk differs from that of the general population; and for researchers, to improve knowledge of cancer etiopathogenesis. This is particularly relevant from a comparative perspective, as dogs and humans share environmental risk exposures that may drive common oncogenic pathways, and share substantial genetic homology (31); therefore, studying breed-related cancer predispositions in dogs may provide clinically meaningful insights for both veterinary and human oncology (32). The present study aims to quantify the differences in the proportion of dogs developing a second primary malignant tumor, compared to the general canine population, to characterize associations among tumor sites and histotypes and to assess the probability of specific histotype co-occurrences, using data collected between 2013 and 2024 from four Italian canine cancer registries: the population-based registries of Lazio, Umbria, and the provinces of Venice and Vicenza, and the hospital-based registry of the University of Pisa.

## 2 Materials and methods

### 2.1 Definition of multiple malignant primary tumors

Multiple malignant primary tumors (mMPTs) were defined, in analogy with the International Association of Cancer Registries (IACR) and the International Agency for Research on Cancer (IARC) rules (1), as the occurrence, in the same dog, of two or more distinct tumor histotypes, either at the same anatomical site or at different sites, regardless of the time of diagnosis. The following criteria were applied to define the tumors included in the study as multiple tumors: (1) each tumor was histologically diagnosed and classified; (2) each tumor was both topographically and histologically distinct; and (3) none of the tumors were metastases. Benign tumors were included only when occurring concurrently with a malignant tumor.

### 2.2 Statistical analysis

#### 2.2.1 Data, inclusion criteria, classification of tumors

Individual records for each dog were obtained from four Italian animal cancer registries (Lazio, Umbria, University of Pisa, and the provinces of Venice and Vicenza), collected in the NILOV database between 2013 and 2024. The initial dataset included information on animal unique identifier, sex, breed, age at first diagnosis, cancer topography, and cancer histotype.

Tumor topography and histotype were coded according to the topographic and morphologic criteria defined in the Vet-ICD-O-Canine-1 coding system (27). Tumor behavior was defined according to the Vet-ICD-O-Canine-1 code suffix: tumors with codes ending in “/0” or “/1” were considered benign, those ending in “/3” malignant. Metastases (i.e., codes ending in “/6”) and *in situ* (i.e., codes ending in “/2”) were excluded from the analysis.

All subjects diagnosed with at least one malignant tumor were included in the study. Therefore, multiple tumors were included both for co-occurrences of malignant tumors and malignant and benign tumors.

#### 2.2.2 Proportional morbidity of mMPTs

In the absence of suitable denominators, proportional morbidity (PM) and corresponding 95% confidence intervals (95% CIs) were used as measures of occurrence. PM was defined as the proportion of cases in a specific tumor category relative to all tumors (e.g., the number of multiple malignant tumors in males divided by the total number of malignant tumors in males). Each category was expressed as a percentage of all tumors, with categories summing to 100%. Proportional morbidity ratios (PMRs) were used to compare groups and time periods (33).

Differences in the proportion of dogs developing second primary malignant tumors relative to the overall canine tumor population were quantified using PMRs and 95% CIs. PMRs were interpreted as follows: a positive association (PMR > 1.0 and 1 not included in the 95% CI) indicated significant over-representation; a negative association (PMR < 1.0 and 1 not included in the 95% CI) indicated under-representation; and no association (PMR = 1.0 or 1 included in the 95% CI) indicated no departure from the expected distribution.

PMRs for multiple malignant primary tumors (mMPTs) were evaluated across age classes (0–3.9, 4–7.9, 8–11.9, and ≥12 years), sex, breed (purebred vs. mixed-breed), and two 6-year calendar periods (2013–2018 and 2019–2024). PMRs were estimated using multilevel mixed-effects Poisson regression models, with subject and registry included as random effects. Because the canine mammary gland is biologically predisposed to developing multiple histologically distinct neoplasms (34), parallel models were fitted including and excluding mammary tumors to ensure that mammary-specific clustering did not obscure broader patterns. Age- and neutering-status–adjusted PMRs were subsequently calculated for each tumor site (Vet-ICD-O-Canine-1 level 0 topography), stratified by sex.

#### 2.2.3 Co-occurrence pattern analysis and associations between tumors

To characterize co-occurrences among tumor sites and histotypes within the same individuals, a network-based co-occurrence analysis was performed on all subjects with multiple primary tumors of which at least one malignant (35). Several co-occurrence analyses were fitted, using different subgrouping strategies of either tumor topography or tumor histotype, aiming to capture both tumor occurrence across anatomical sites and associations among histotypes independent of site.

The co-occurrence pattern of analysis was assessed either by tumor site (Vet-ICD-O-Canine-1 level 0 topography) or by tumor histotype (Vet-ICD-O-Canine-1, level 2).

First, contingency matrices for the variable of interest were generated, where each dog represented a scenario (rows) and the variable of interest occurrences represented events (columns). Similarity between co-occurrences was assessed using Haberman’s normalized residuals. The resulting adjacency matrix was converted into a network in which nodes represented the variable of interest (i.e the tumor site or the histotype) and edges represented the similarity between two possible events. Graphical networks representations were produced in which node size reflected degree centrality (i.e., number of significant connections), label size indicated the frequency of occurrence, and the edge width is proportional to Haberman’s normalized residuals. Positive Haberman’s residuals indicates positive connection between two nodes (<2 not relevant, 2–3 weak, >3 strong), negative Haberman’s residuals indicates negative connection. Events were then grouped according to their co-occurrence patterns using the walktrap cluster detection method which exploits the principle that random walks on a network tend to become “trapped” in dense, well-connected areas (36). These areas correspond to the communities, or clusters, of the variable of interest.

Finally, the probability of associations between tumor histotypes (Vet-ICD-O-Canine-1, level 2) for multiple primary tumors of which at least one malignant was assessed using the *χ*^2^-test, using different subgrouping strategies, aiming to capture both tumor occurrence across anatomical sites and associations among histotypes independent of site.

Network co-occurrence analysis was performed using the netCoin R package (https://cran.r-project.org/web/packages/netCoin/vignettes/netCoin.html, accessed on September 2025). Data management and all other statistical analyses were performed using STATA 19.5 (StataCorp, TX).

## 3 Results

Individual canine records (*n* = 16,618) were obtained from the four selected Italian animal cancer registries (3,728 subjects from Lazio, 1,938 from Umbria Region, 4,604 from the University of Pisa, and 6,384 from the provinces of Venice and Vicenza) and collected in the NILOV database between 2013 and 2024.

### 3.1 Proportional morbidity of mMPTs

mMPTs were identified in 11.3% of dogs, with some variation across registries: Lazio 8.5%, Umbria 20%, Venice-Vicenza 15.2%, University of Pisa 4.3%. Table 1 shows the distribution of individuals included in the study by sex, neutering status, age class and breed. The age distribution of dogs included in the study is reported in Supplementary Figure 1.

**Table 1:** Dogs involved in the study, frequency and proportion by sex, neutering status and age class.

Parameter		Number of subjects	Proportion	95%CI
Sex and neutering staus				
Male	Neutered	1,083	17%	16–17%
	Intact	5,245	80%	79–81%
	Neutering status not available	214	3%	3–4%
	Total	6,542	40%	39–40%
Female	Neutered	3,146	32%	31–33%
	Intact	3,727	38%	37–39%
	Neutering status not available	3,050	31%	30–32%
	Total	9,923	60%	60–61%
	Sex and neutering status not available	153	1%	0.8–1.1%
Age class				
	0–3.9y	437	3%	2–3%
	4–7.9y	2,959	19%	18–19%
	8–11.9y	7,309	46%	45–47%
	>=12y	5,196	33%	32–33%
	Total	15,901		
	Age not available	717	5%	4–5%

Two proportional morbidity ratio (PMR) models, differing only by the inclusion (Model A) or exclusion (Model B) of mammary gland tumors, were used to evaluate potential proportional occurrence modifiers for multiple primary tumors (mMPTs) (Table 2). In both models, females showed a proportional occurrence of mMPTs moderately higher than males (Model A: PMR 1.3, 95% CI 1.2–1.5; Model B: PMR 1.3, 95% CI 1.1–1.4). Proportional occurrence was also moderately higher in intact subjects when compared to neutered ones (both models: PMR = 1.4, 95%CI 1.2–1.6). Age demonstrated the strongest gradient, with a clear increase in proportional occurrence across all successive age classes. Compared with dogs aged 0–3.9 years, those aged 4–7.9 years exhibited a threefold increase (Model A:PMR 3.4, 95% CI 1.7–6.8, Model B:3.8, 95%CI 1.7–8.3), which rose further in the 8–11.9 years group (Model A: PMR 5.8, 95% CI 2.9–11.6; Model B: PMR 6.32, 95% CI 2.9–13.7) and peaked in dogs ≥12 years (Model A: PMR 7.3, 95% CI 3.7–14.6; Model B: PMR 7.7, 95% CI 3.6–16.9). Purebred status (referred as “Breed: any breed”) showed only a borderline effect with both models when compared to mixed breed (referred as “Breed: no breed”). Overall, results from Models A and B were highly consistent, highlighting sex and age as the most relevant factors for mMPTs occurrence.

**Table 2:** Results of multilevel mixed-effect Poisson regression models (random effects: subject and registry), with and without mammary gland tumours.

Independent variable	Model A (mammary gland included)		Model B (mammary gland excluded)	
	PMR	95% CI	PMR	95% CI
Sex: Male	1.0	–	1.0	–
Sex: Female	1.3	1.2–1.5	1.3	1.1–1.4
Neutered/spayed	1.0	–	1.0	–
Intact	1.4	1.2–1.6	1.4	1.2–1.6
Age: 0–3.9y	1.0	–	1.0	–
Age: 4–7.9y	3.4	1.7–6.8	3.8	1.7–8.3
Age: 8–11.9y	5.8	2.9–11.6	6.3	2.9–13.7
Age: > = 12y	7.3	3.7–14.6	7.7	3.6–16.9
Breed: no breed	1.0	–	1.0	–
Breed: any breed	1.2	1–1.3	1.2	1–1.3
Period:2013–2018	1.3	–	1.0	–
Period:2019–2024	0.8	0.7–1	0.7	0.6–0.9

PMR = proportional morbidity ratio.

**Figure 1:**
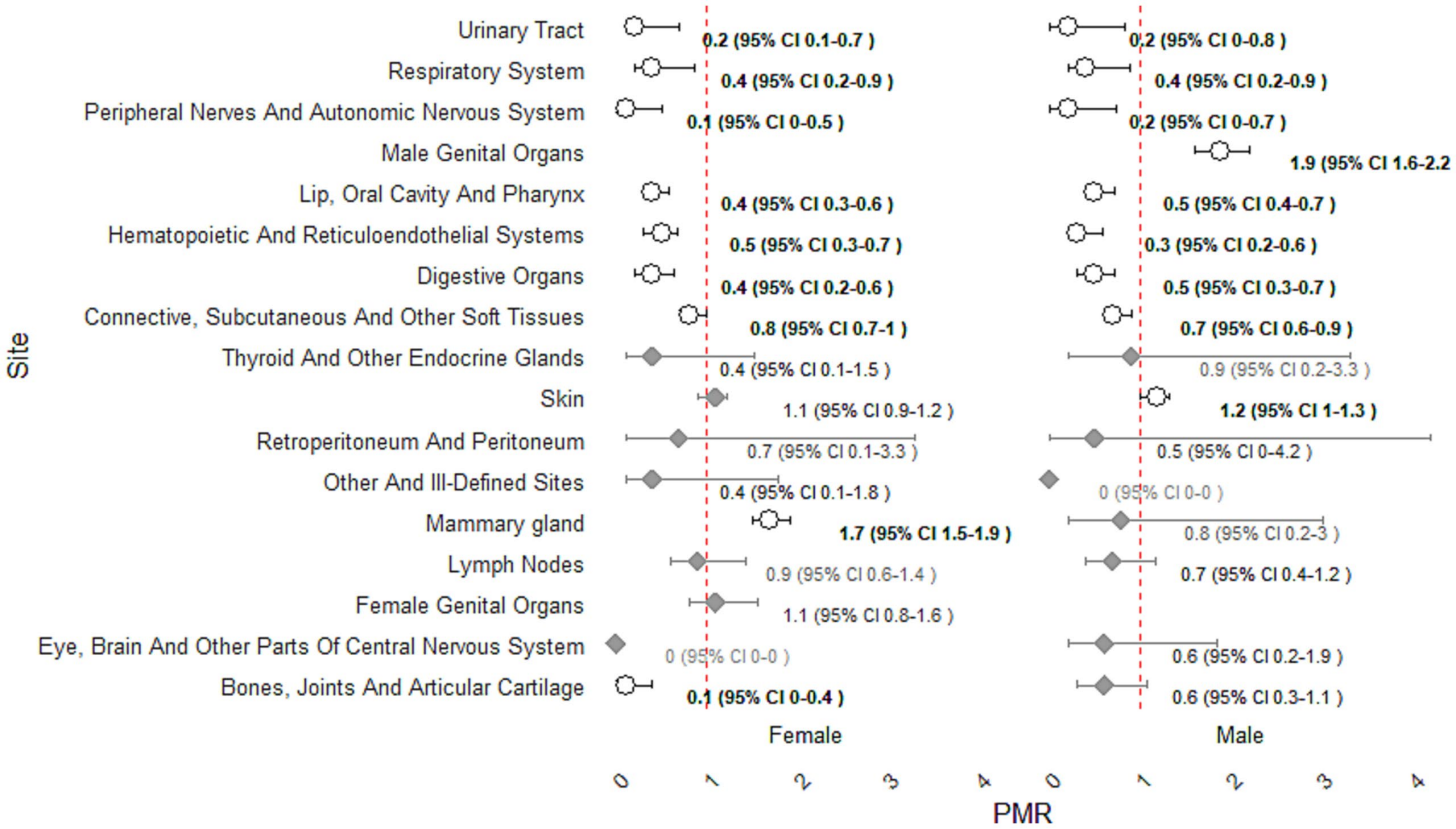
Proportional morbidity ratios (PMRs) and 95% CIs for developing multiple malignant primary tumors by anatomical site and sex. A vertical dashed line indicates PMR = 1. Circles and bold values represent (statistical) significant PMRs; diamonds and plain values represent non-significant PMRs. Upper confidence limits >10 were truncated.

Across both sexes, significantly reduced proportional occurrence was observed for tumors of the lip, oral cavity and pharynx, digestive organs, hematopoietic tissues, connective/soft tissues, peripheral nerves, bones and joints, and urinary tract. In contrast, male genital organs and the mammary gland (in females) exhibited higher proportional occurrence of mMPTs (PMR 1.9, 95% CI 1.6–2.2; and PMR 1.7, 95% CI 1.5–1.9, respectively). Lymph nodes showed values close to unity, indicating no meaningful differences in proportional occurrence. Several other sites, such as thyroid, retroperitoneum, and central nervous system, had wide confidence intervals overlapping 1 (Figure 1).

The proportional occurrence profile also varied significantly by tumor histotype at level 2 group of vet-ICD-O canine. In males, lipomatous neoplasms (PMR 2.1, 95% CI 1.5–3.1), basal cell neoplasms (PMR 1.8, 95% CI 1.1–2.8), specialized gonadal neoplasms (PMR 1.8, 95% CI 1.5–2.1) and germ cell neoplasms (PMR 1.3, 95% CI 1.1–1.6) showed the highest proportional occurrence. In females, tumors belonging to the group adenomas and adenocarcinomas (PMR 1.5, 95% CI 1.3–1.7), adnexal and skin appendage neoplasms (PMR 1.3, 95%CI 1.03–1.6), basal cell neoplasms (PMR 2.2, 95% CI 1.6–3.0), epithelial neoplasms (PMR 1.6, 95% CI 1.3–1.9), lipomatous neoplasms (PMR 2.3, 95% CI 1.7–3.0) and odontogenic tumors (PMR 3.5, 95%CI 1.2–10.6) were most strongly associated with mMPTs. In both sexes, soft tissue tumors and sarcomas, squamous cell neoplasms, Hodgkin and non-Hodgkin lymphomas, nerve sheath tumors, osseous and chondromatous neoplasms showed, instead, significantly reduced proportional occurrences. In females only, additional reduced proportional occurrences of developing mMPTs were observed for mast cell neoplasms, melanocytoma and melanomas, and transitional cell papillomas and carcinomas.

### 3.2 Co-occurrence pattern analysis

Several co-occurrence patterns were identified and analyzed based on both tumor topography and histotype.

**Figure 2:**
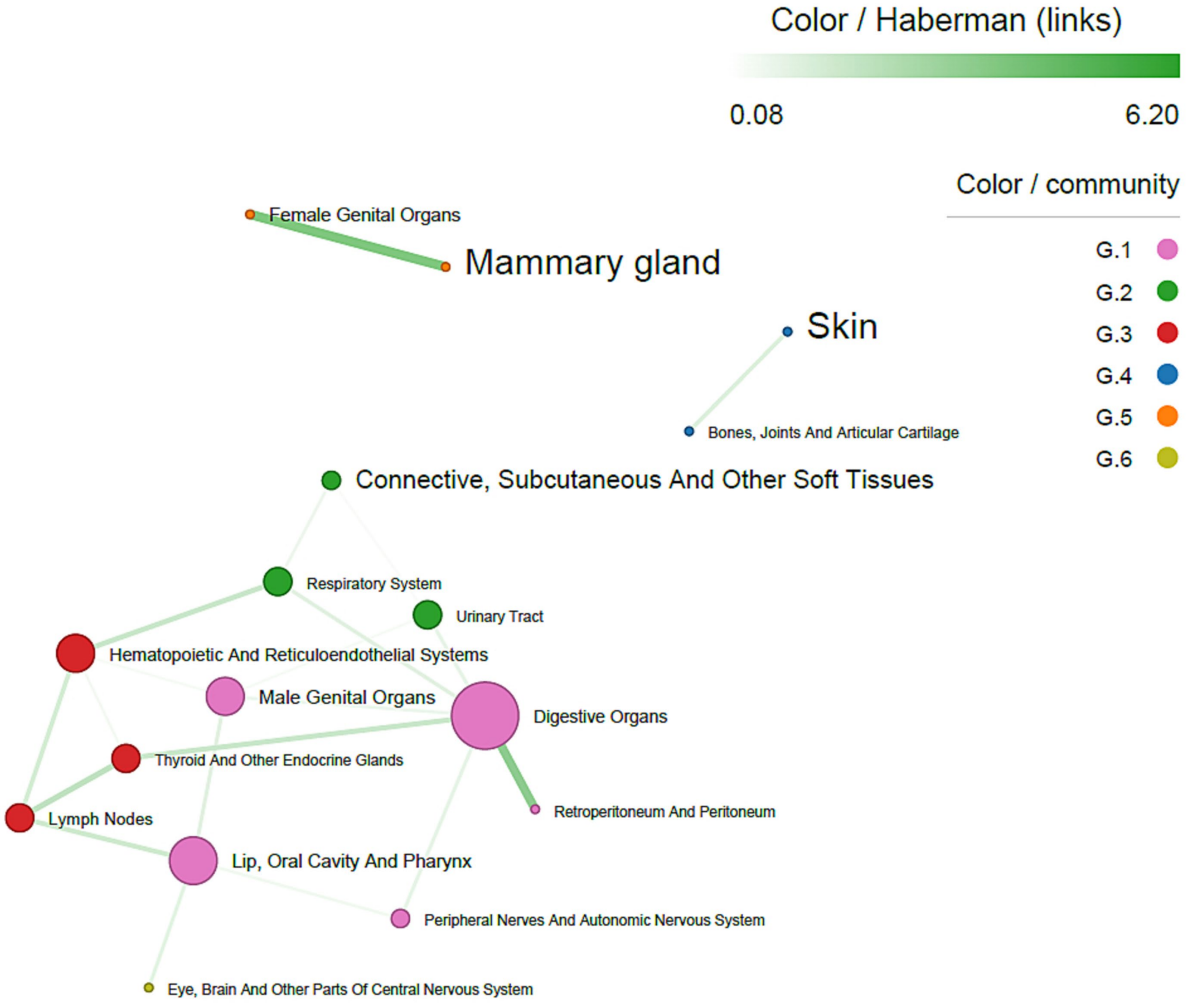
Co-occurrence network of tumor sites, in both sexes. The size of nodes is proportional to centrality degree; label size is proportional to frequency of occurrence. Nodes (tumor sites) belonging to the same community have the same color. Edge width is proportional to Haberman’s residuals.

Topographic co-occurrence analysis of mMPTs showed that tumors of the mammary gland most frequently co-occurred with skin tumors. In contrast, tumors of the digestive organs were the most central nodes in the network, followed by tumors of the male genital organs and those of the hematopoietic and reticuloendothelial systems. Walktrap community detection identified five communities, three of which were interconnected and encompassed most tumor sites. Tumors of the mammary gland and female genital organs formed a distinct community, as did tumors of the skin and bones, joints, and articular cartilage. All site-to-site associations were positive. Moderate associations were observed between lymph nodes and thyroid and other endocrine glands (Haberman’s residuals = 2.31), whereas strong associations were found between digestive organs and retroperitoneum/peritoneum (Haberman’s residuals = 3.96) and between mammary gland and female genital organs (Haberman’s residuals = 3.97) (Figure 2).

**Figure 3:**
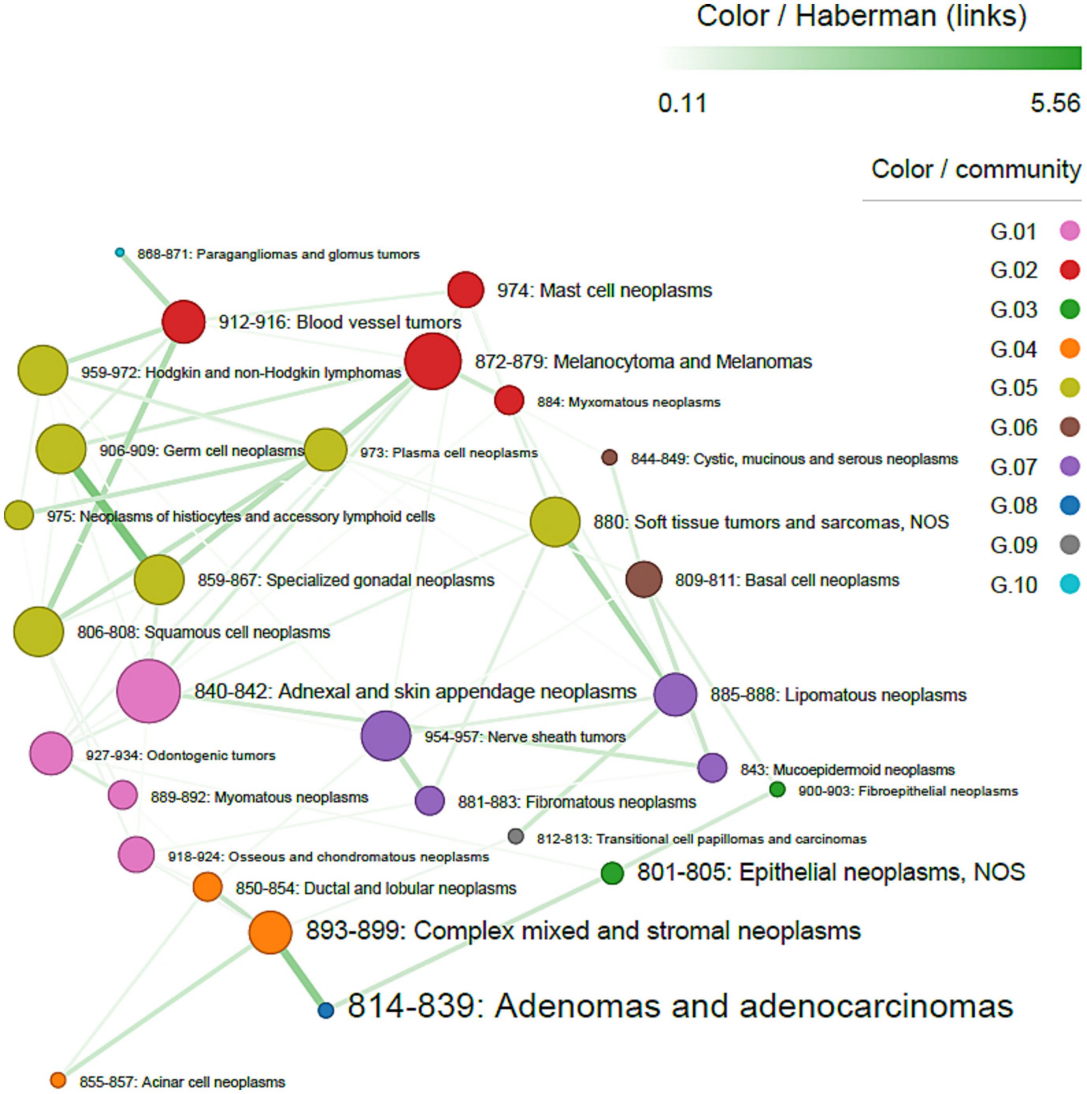
Co-occurrence network of tumor histotypes, in both sexes. The size of nodes is proportional to centrality degree; label size is proportional to frequency of occurrence. Nodes (tumor sites) belonging to the same community have the same color. Edge width is proportional to Haberman’s residuals.

Histotype co-occurrence analysis, including both sexes and considering only pairs with at least one malignant tumor, showed that adenomas/adenocarcinomas most frequently co-occurred with complex mixed and stromal neoplasms. However, adnexal and skin appendage neoplasms were the most central histotypes in the network, followed by melanocytomas and melanomas. Walktrap identified 10 interconnected communities, with adenomas/adenocarcinomas forming a single distinct community. All associations were positive, with strong connections between specialized gonadal neoplasms and germ cell neoplasms (Haberman’s residuals = 5.56), adenomas/adenocarcinomas and complex mixed and stromal neoplasms (Haberman’s residuals = 4.74), soft tissue tumors and sarcomas and lipomatous neoplasms (Haberman’s residuals = 3.47), and blood vessel tumors and squamous cell neoplasms (Haberman’s residuals = 3.28) (Figure 3).

**Figure 4:**
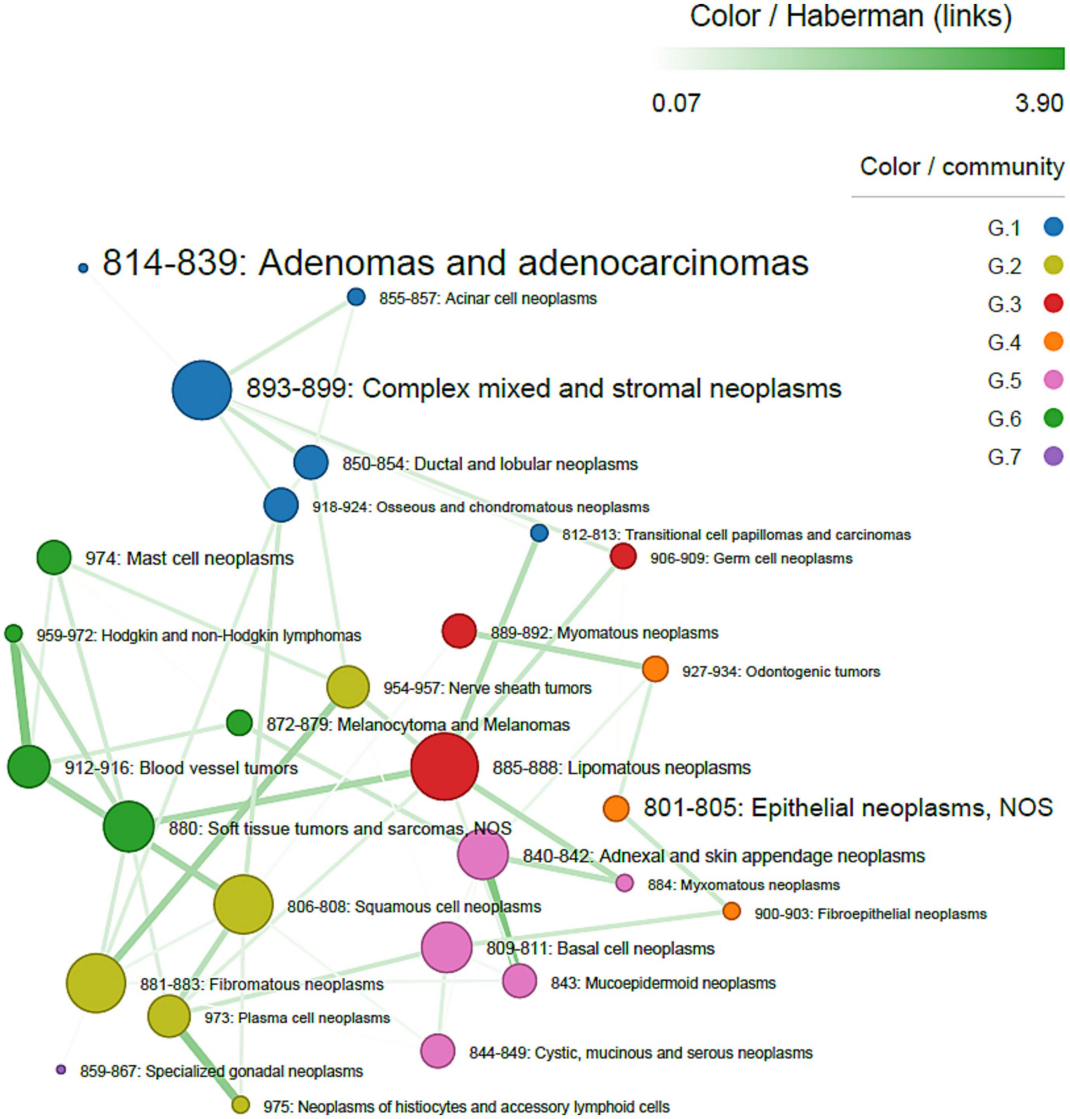
Co-occurrence network of tumor histotypes, in females. The size of nodes is proportional to centrality degree; label size is proportional to frequency of occurrence. Nodes (tumors) belonging to the same community have the same color. Edge width is proportional to Haberman’s residuals.

**Figure 5:**
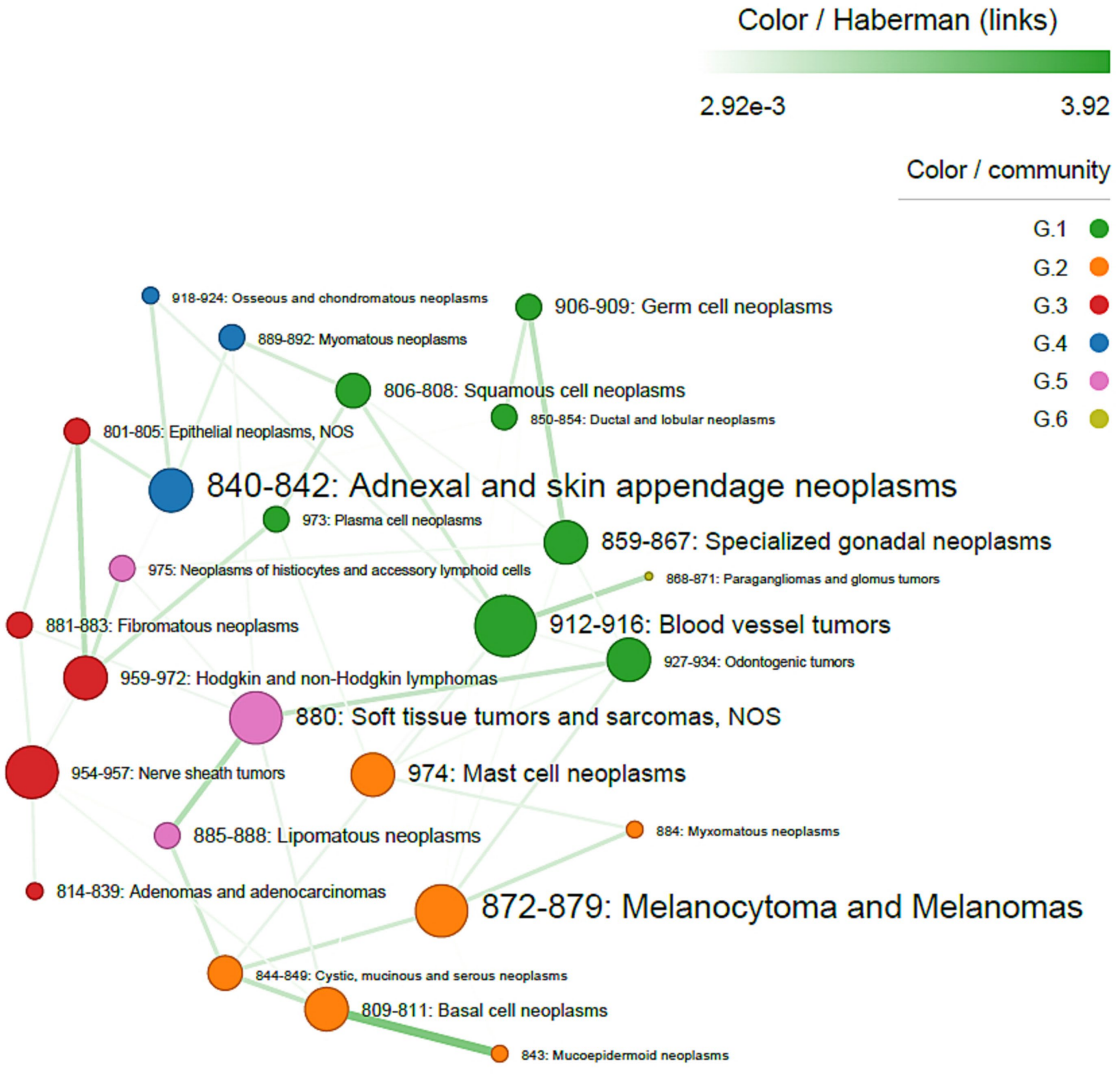
Co-occurrence network of tumor histotypes, in males. The size of nodes is proportional to centrality degree; label size is proportional to frequency of occurrence. Nodes (tumors) belonging to the same community have the same color. Edge width is proportional to Haberman’s residuals.

Sex-stratified analyses revealed different patterns. In females, adenomas/adenocarcinomas were the most frequent histotypes, whereas lipomatous neoplasms were the most central. Strong associations were observed between Blood vessel tumors and Hodgkin and non-Hodgkin lymphomas (Haberman’s residuals = 3.90), adnexal and skin appendage neoplasms and mucoepidermoid neoplasms (Haberman’s residuals = 3.73), and neoplasms of histiocytes and accessory lymphoid cells and plasma cell neoplasms (Haberman’s residuals = 3.40) (Figure 4). In males, adnexal and skin appendage neoplasms were the most frequent histotypes, while blood vessel tumors were the most central. The strongest association was observed between basal cell neoplasms and mucoepidermoid neoplasms (Haberman’s residuals = 3.92) (Figure 5).

### 3.3 Associations between tumors

**Figure 6:**
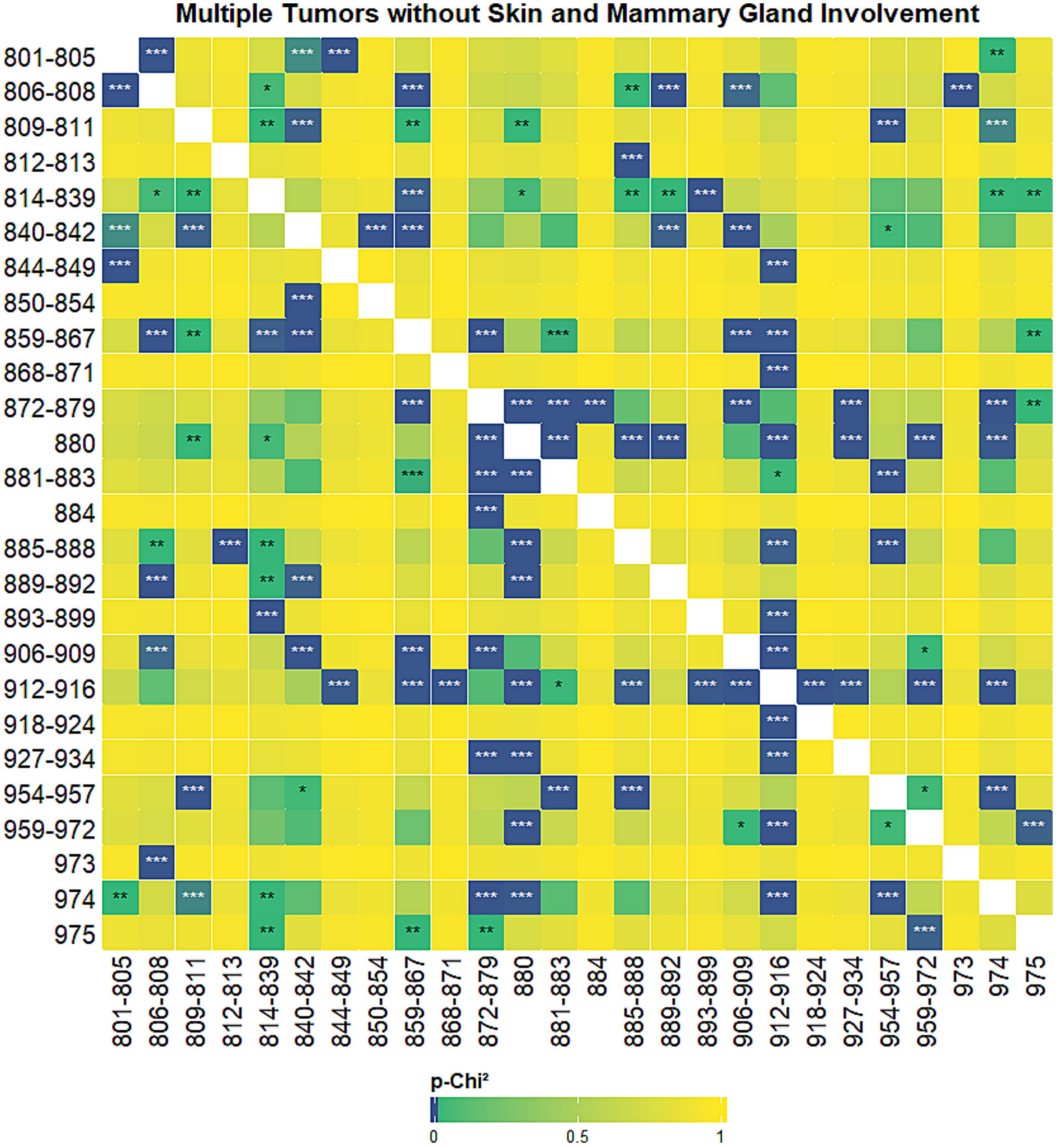
Heatmap showing univariate probability of association (*p*-*χ*^2^) between tumor histotypes, in both sexes, after the exclusion of tumors of the mammary gland and skin. High probability of association (lowest *p*-*χ*^2^ values) are displayed with darker colors, the color gradient degrades towards yellow and then towards green starting from *p*-*χ*^2^ values above 0.001. ***p-*χ*^2^ values < 0.001; **p-*χ*^2^ values < 0.01;*p-*χ*^2^ values < 0.05. Tumor hystotypes are identified using their Vet-ICD-O-Canine-1, level 2 code (see Supplementary Table 1).

**Figure 7:**
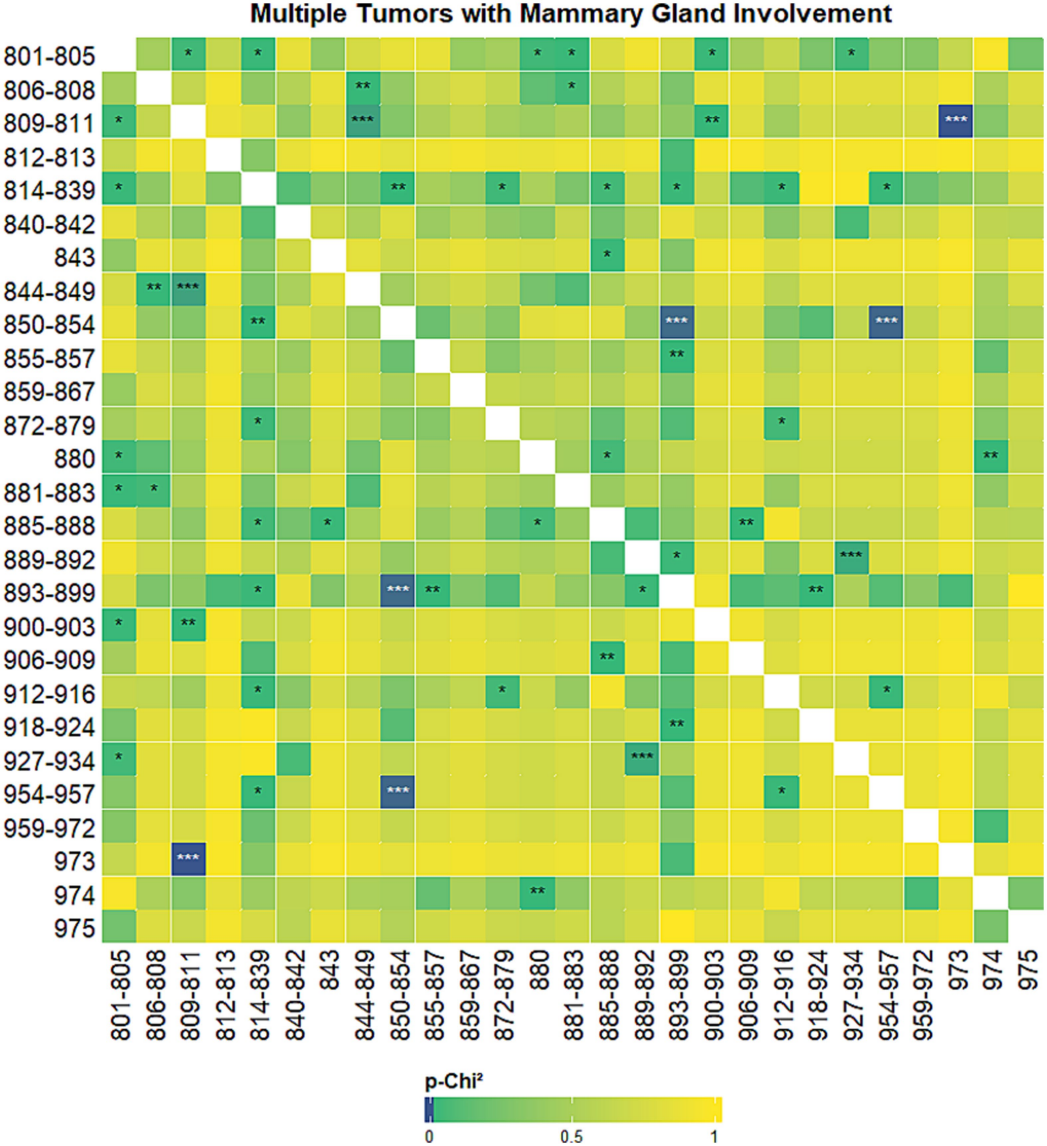
Heatmap showing univariate probability of association (*p*-*χ*^2^) between tumor histotypes when mammary gland was involved. High probability of association (lowest *p*-*χ*^2^ values) are displayed with darker colors, the color gradient degrades towards yellow and then towards green starting from *p*-*χ*^2^ values above 0.001. ***p-*χ*^2^ values < 0.001; **p-*χ*^2^ values < 0.01;*p-*χ*^2^ values < 0.05. Tumor hystotypes are identified using their Vet-ICD-O-Canine-1, level 2 code (see Supplementary Table 1).

**Figure 8:**
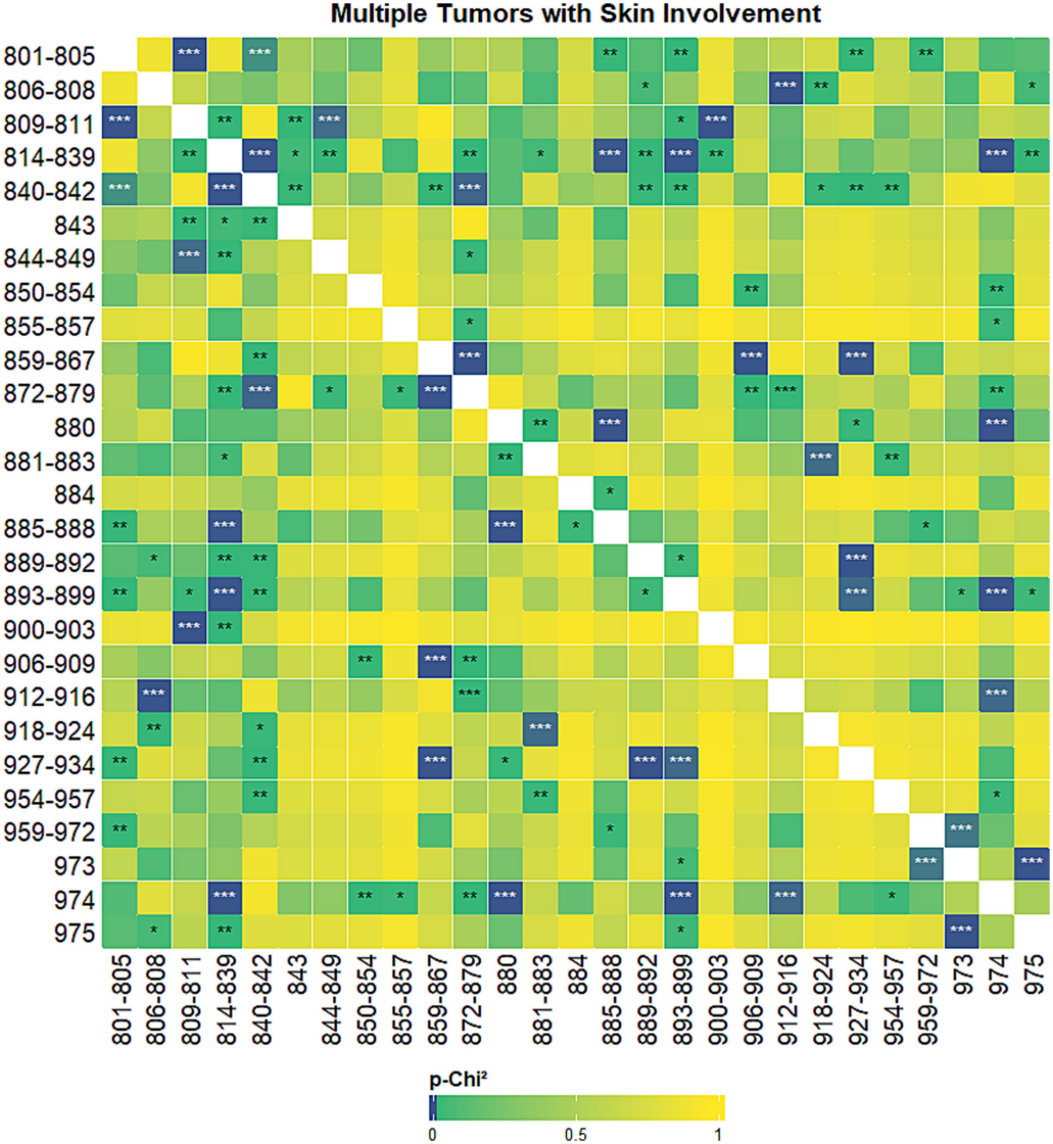
Heatmap showing univariate probability of association (*p*-*χ*^2^) between tumor histotypes when skin was involved. High probability of association (lowest *p*-*χ*^2^ values) are displayed with darker colors, the color gradient degrades towards yellow and then towards green starting from *p*-*χ*^2^ values above 0.001. ***p-*χ*^2^ values < 0.001; **p-*χ*^2^ values < 0.01;*p-*χ*^2^ values < 0.05. Tumor hystotypes are identified using their Vet-ICD-O-Canine-1, level 2 code (see Supplementary Table 1).

The results of the co-occurrence analysis prompted us to seek for univariate probability of association (*p*-*χ*^2^) between tumor histotypes, using different grouping strategies. We first analyzed the probability of associations between histotypes excluding both the mammary gland and the skin (Figure 6), then we focused on the probability of associations between histotypes when the mammary gland was involved (Figure 7) and, finally, on the probability of association when the skin was involved (Figure 8).

As shown in Figure 6 after the exclusion of tumors of the mammary gland and of the skin, blood vessel tumors showed the highest number of very significant association with other histotypes, followed by soft tissue tumors and sarcomas, melanocytoma and melanomas and specialized gonadal neoplasms. Epithelial neoplasms, adenomas and adenocarcinomas have a low number of very significant associations (*p* < 0.001).

When analysing the univariate association between histotypes of multiple tumors when at least one of the tumors was localised in the mammary gland we could see that very significant associations (*p* < 0.001) were seen only between basal cell neoplasms and plasma cell neoplasms, ductal and lobular neoplasms and complex mixed and stromal neoplasms, ductal and lobular neoplasms and nerve sheath tumors, myomatous neoplasms and odontogenic tumors (Figure 7).

When analysing the univariate association between histotypes of multiple tumors when at least one of the tumors was localised in the skin, we found very significant associations between mast cell tumor and adenomas and adenocarcinomas, soft tissue tumors and sarcomas, complex mixed and stromal neoplasms, blood vessel tumors. Conversely, mucoepidermoid neoplasms, cystic, mucinous and serous neoplasms, ductal and lobular neoplasms, acinar cell neoplasms, myxomatous neoplasms, fibroepithelial neoplasms and nerve sheath tumors show no highly significant associations, when the skin is involved (Figure 8).

## 4 Discussion

This study confirms that, as in humans, dogs frequently develop multiple primary tumors (mMPTs) with consistent patterns of proportional occurrence across sexes, age and histotypes (6–10). Although this finding is not new, few work are available on the subject in literature. Moreover, due to different study design, differences in criteria of inclusion and possible differences in tumor classification it is difficult to compare our results in the context of existing literature.

The definition of a second primary tumor is crucial, yet the criteria vary among studies. In human oncology, two main classification systems are commonly used: the Surveillance, Epidemiology, and End Results (SEER) Program rules, widely adopted in North America (37) and the IACR/IARC rules, used internationally (1). SEER criteria consider histology, site, laterality, and the time interval from the initial diagnosis, while the IARC/IACR rules are more restrictive, registering only one tumor per organ unless it is histologically distinct. In our study, most tumors were detected simultaneously, making the SEER definition inappropriate and preventing temporal analyses. Consequently, the temporal dynamics of tumor associations could not be assessed, hampering the identification of tumor histotypes as potential risk factors for other second primary tumors.

Sex and age consistently emerged as the principal determinants of increased mMPT proportional occurrence. Similarly to what was reported by Bender and coll. (7), females in our study exhibited a modest but consistent excess in mMPTs proportional occurrence compared with males, while age showed a strong, progressive effect, with proportional occurrence increasing steadily from mid-life into older age. This pattern likely reflects cumulative biological processes that become more pronounced with aging. Being intact also appeared to increase the proportional occurrence of mMPTs; however, this finding should be interpreted with caution, as nearly all males in the dataset were intact, and the timing of neutering was not reported and may have coincided with tumor removal. Although several breeds have been reported to be overrepresented in the development of specific tumor types (38, 39), our findings—consistent with those of Rebhun and colleagues (8)—indicate that breed contributed only marginally to the proportional occurrence. This suggests that genetic background or selective breeding practices may have a limited influence on the occurrence of multiple primary tumors. Although we attempted to explore the data by individual breed, the number of subjects within each breed was insufficient to obtain robust or reliable results. Moreover, our analysis was not specifically designed to evaluate differences in the occurrence of mMPTs among individual breeds.

Differences observed between purebred and mixed-breed dogs should therefore be interpreted with caution. In our model, all purebred dogs were grouped into a single category, despite representing diverse breeds with heterogeneous predispositions to various cancers. This approach may have masked potential breed-specific susceptibilities to multiple primary tumors. Future studies incorporating genomic, environmental, and lifestyle data are warranted to better elucidate possible breed-related cancer risks.

Registry-specific differences were also observed. The data registered by the University of Pisa showed the lowest proportion of mMPTs (4.3%), whereas the Umbria registry reported the highest (20%). These discrepancies may reflect regional specialisation in certain tumor types, differences in referral patterns, or variable registry completeness. Such heterogeneity was accounted for, incorporating the registry-specific random effect in the model, to minimize potential bias.

The slight decrease in proportional occurrence observed in 2019–2024 likely reflects changes in diagnostic activity or improvements in tumor registration, rather than a true temporal shift.

The canine mammary gland is biologically predisposed to developing multiple, histologically distinct neoplasms (31). For this reason, we applied two proportional morbidity ratio (PMR) models that differed only in the inclusion (Model A) or exclusion (Model B) of mammary gland tumors, in order to verify that mammary-specific clustering did not mask broader epidemiological patterns. Overall, the consistency of the two models supports the robustness of the analysis and confirms sex and, above all, age as the primary drivers of mMPT occurrence.

Site-specific analyses revealed a heterogeneous distribution of proportional occurrence of tumors across anatomical locations. Several sites showed a consistently reduced proportional occurrence, in contrast, marked excess proportional occurrence was identified for the genital system, particularly the male genital organs (PMR 1.9, 95% CI 1.6–2.2). Female genital organs did not show a comparable increase, but the mammary gland exhibited a significant excess proportional occurrence in females (PMR 1.7, 95% CI 1.5–1.9). Other sites, displayed wide confidence intervals overlapping unity, reflecting limiting interpretability due to low case numbers. Notably, the skin showed estimates close to or slightly above unity. The proportional occurrence of mMPTs varied markedly according to histotype (Vet-ICD-O level 2) and showed clear sex-specific patterns.

Overall, these results indicate that the proportional occurrence of multiple primary tumors is unevenly distributed across organs and concentrated in specific anatomical systems—particularly the male genital tract and the female mammary gland—while other systems appear consistently underrepresented. These differences -in line with what previously reported by Bender and coll. (7)—likely reflect a combination of biological susceptibility, diagnostic practices, and exposure profiles.

The network analysis shows that strong connections exist between mammary and female genital organ tumors, whereas there is a lack of connection between tumors in other sites. This finding supports what previously reported by Bender and coll. (7) and highlight the strong multiple primary potential for the combination of mammary gland and female genital organs tumors, suggesting that those tumor sites are on the same oncogenic pathway, as seen for instance for BRCA mutation in human breast and ovarian cancers (40, 41).

The centrality of adnexal and skin appendage neoplasms within the overall network indicates that cutaneous tumors may represent a key hub in the development of multiple primary tumors. The over representation within multiple distinct malignancies of mast cell tumors and malignant melanoma was previously reported by Rebhun and coll. (8). Although this could suggest the existence of a high exposure of the skin to environmental carcinogens, or intrinsic biological susceptibility, these results should be interpreted with caution, as mast cell tumors are the most common malignant skin tumors in dogs (42); therefore, the high proportion of multiple tumors may reflect incidental diagnoses made during evaluation or treatment for a different neoplasm.

The strong association between specialized gonadal neoplasms and germ cell tumors is biologically plausible, as these entities arise within the same anatomical and developmental context, potentially sharing hormonal and genetic drivers (42). Further analyses are needed to identify the drivers of other highly significant associations.

Sex-stratified analyses revealed distinct network configurations, underscoring the role of sex-related biological factors. However, these findings should be interpreted as descriptive rather than causal. High-degree nodes may represent histotypes with broader systemic predispositions (e.g., tissue tropism, hormonal influence) or shared etiological mechanisms/drivers.

Overall, these findings support the concept that multiple primary tumors in dogs are not randomly distributed but follow structured biological patterns, potentially shaped by tissue origin, hormonal status, immune function, and environmental exposure. Further longitudinal and molecular investigations are needed to clarify the causal mechanisms underlying these associations.

Several limitations should be acknowledged. First, network structure may be influenced by the relative frequency of common tumor types, potentially exaggerating their connectivity. Second, sex-related differences in sample size may bias the apparent centrality of certain histotypes. Despite these limitations, graphical networks provide a valuable, intuitive representation of co-occurrence patterns and a foundation for further mechanistic investigation.

Multiple highly significant associations were identified between histotypes occurring within the same individual, suggesting potential shared etiological pathways or diagnostic convergence among related tumor classes. In the present analysis, we deliberately focused on associations with *p*-values < 0.001 in order to highlight the most robust and biologically meaningful links. However, numerous additional associations reached statistical significance at less stringent thresholds, and these should be taken into account in future investigations to provide a more comprehensive understanding of tumor co-occurrence patterns.

After excluding mammary gland and skin tumors, blood vessel tumors emerged as the histotype with the highest number of very significant associations, this result, in line with what previously reported by Rehbun and coll. (7), suggests a potentially central biological role in the development of multiple primary tumors beyond the two most common anatomical sites. The prominent position of soft tissue sarcomas, melanocytic tumors, and specialized gonadal neoplasms further supports the hypothesis that mesenchymal and pigment-cell lineages may share susceptibility pathways or systemic predisposing factors. In contrast, epithelial neoplasms such as adenomas and adenocarcinomas showed few very significant associations, suggesting a more site-specific or less interconnected pattern of occurrence in this context.

When at least one tumor was localized in the mammary gland, only a limited number of very significant associations were observed. The link between ductal and lobular neoplasms and complex mixed and stromal tumors is biologically plausible, given their shared mammary origin and epithelial–stromal interactions. Associations with nerve sheath, odontogenic, and myomatous tumors may reflect either shared developmental pathways or incidental co-occurrence, but their restricted number suggests that mammary involvement does not broadly drive multiple strong histotype pairings.

In contrast, when the skin was involved, mast cell tumors showed multiple very significant associations with diverse histotypes, including adenomas/adenocarcinomas, soft tissue sarcomas, complex mixed tumors, and vascular neoplasms. This pattern may suggest a heightened biological interplay between cutaneous immune-mediated processes and other tumor types, although the high prevalence of skin tumors could also increase the probability of coincidental detection. The absence of strong associations with several epithelial and mesenchymal subtypes suggests that not all tumor lineages are equally influenced by cutaneous involvement.

Overall, these findings reinforce the idea that the anatomical context significantly shapes histotype co-occurrence patterns, with vascular and mesenchymal tumors playing a particularly interconnected role outside mammary and cutaneous sites.

## 5 Conclusion

This study confirms that, similarly to humans, dogs frequently develop multiple primary malignant tumors (mMPTs), and that their occurrence follows structured and non-random patterns. Despite methodological heterogeneity in the literature and differences in tumor classification systems, our findings consistently identify sex and age as the main determinants of mMPT occurrence. Females showed a modest but persistent excess proportional occurrence compared with males, while age exerted a strong cumulative effect, supporting the role of progressive biological alterations over time.

Anatomical distribution was uneven, with a marked concentration of mMPTs in the male genital tract and in the female mammary gland, whereas several other systems appeared underrepresented. These differences likely reflect a combination of intrinsic biological susceptibility, hormonal influences, and diagnostic practices. The strong network connection between mammary and female genital tumors further supports the existence of shared oncogenic pathways, potentially analogous to hormone-related cancer syndromes described in human oncology.

Network analyses demonstrated that tumor co-occurrence is not random but organized in biologically meaningful clusters. Sex-stratified analyses revealed distinct architectures, reinforcing the influence of hormonal and biological context in shaping tumor associations.

Although breed contributed only marginally, the broad categorization of purebred dogs may have masked breed-specific susceptibility. Registry heterogeneity and the lack of temporal information—due to the simultaneous detection of most tumors and differences in second primary tumor definitions—limit causal interpretation and prevent assessment of temporal sequences.

Overall, these findings indicate that mMPTs in dogs arise within structured biological frameworks influenced by sex, age, tissue origin, and possibly hormonal and immune-related mechanisms. Future longitudinal, genomic, and environmental studies are needed to clarify causal pathways and to improve surveillance, prevention, and clinical management strategies in canine oncology.

## Data Availability

The datasets presented in this article are not readily available because the data is the property of the NILOV network and are accessible upon reasonable request, subject to authorization by the network. Requests to access the datasets should be directed to cerovec@izsplv.it.
