# Supplementary figures and images for: Multiple malignant primary tumors in dogs: an Italian registry-based epidemiological study
Source: Front Vet Sci. 2026 May 5;13:1755025. doi: 10.3389/fvets.2026.1755025 (PMC13185691; doi:10.3389/fvets.2026.1755025)

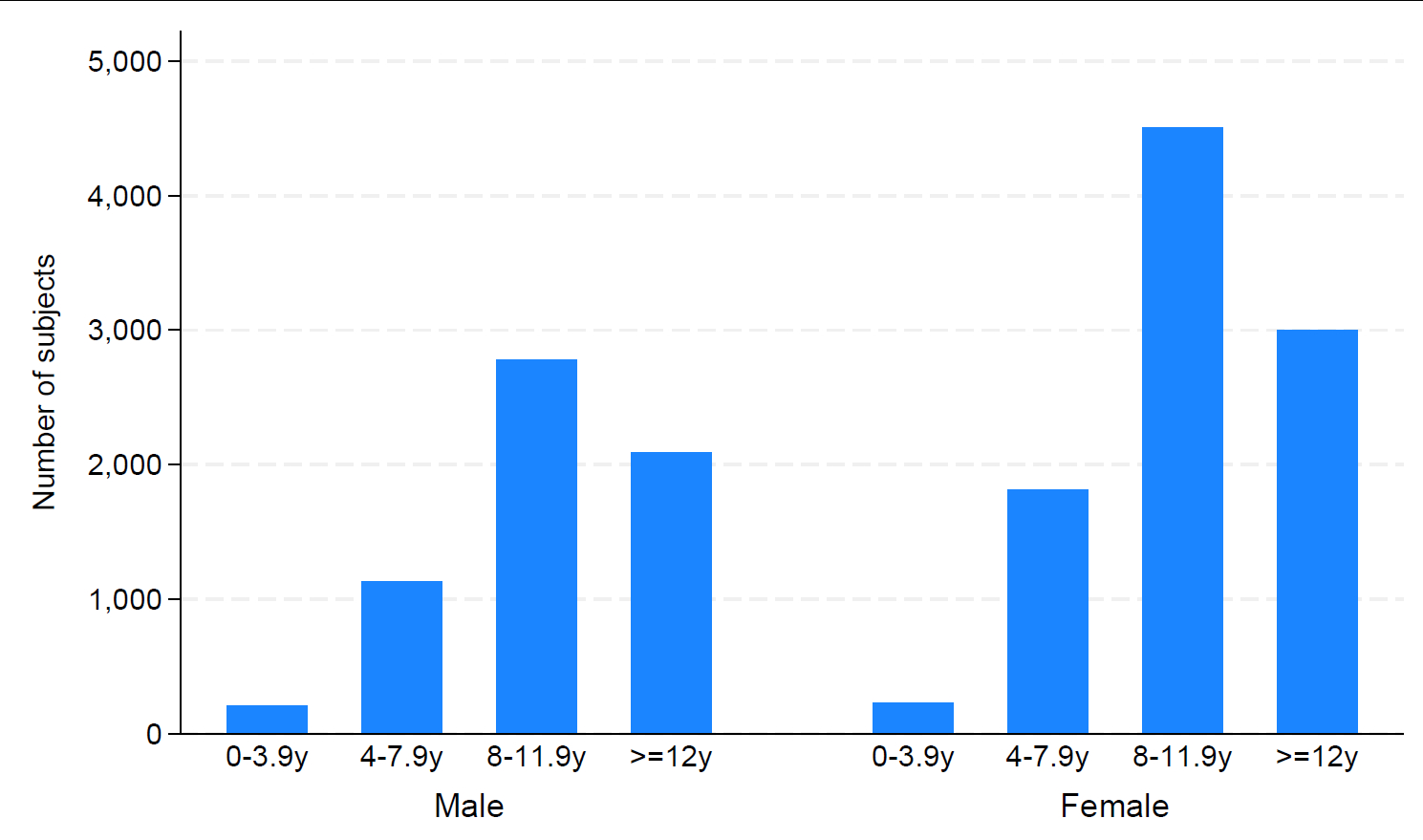

Supplement: Supplementary file 1 [file Image_1.JPEG]
